# Personalized Reminders for Immunization Using Short Messaging Systems to Improve Human Papillomavirus Vaccination Series Completion: Parallel-Group Randomized Trial

**DOI:** 10.2196/26356

**Published:** 2021-12-27

**Authors:** Chelsea S Wynn, Marina Catallozzi, Chelsea A Kolff, Stephen Holleran, Dodi Meyer, Rajasekhar Ramakrishnan, Melissa S Stockwell

**Affiliations:** 1 Division of Child and Adolescent Health Department of Pediatrics Columbia University Irving Medical Center New York, NY United States; 2 Department of Population and Family Health Mailman School of Public Health Columbia University Irving Medical Center New York, NY United States; 3 NewYork-Presbyterian Hospital New York, NY United States

**Keywords:** text messaging, mobile reminders, human papillomavirus, adolescent, text reminders, vaccine completion, vaccine decision-making, vaccine education, transtheoretical model, mobile phone, smartphone, mHealth, mobile health, minority health

## Abstract

**Background:**

Completion rates among adolescents who initiate the human papillomavirus (HPV) vaccine 3-dose series are low. SMS text message vaccine reminders are effective, but less is known about the best types for HPV series completion or the ability to assess and target vaccine decision-making stage.

**Objective:**

The aim of this study is to compare the effectiveness of HPV vaccine series completion in minority adolescents who received precision and educational versus conventional SMS text message reminders.

**Methods:**

Enrolled parents of adolescents aged 9-17 years who received the first HPV vaccine dose at 1 of the 4 academic-affiliated community health clinics in New York City were randomized 1:1 to 1 of the 2 parallel, unblinded arms: precision SMS text messages (which included stage-targeted educational information, next dose due date, and site-specific walk-in hours) or conventional SMS text messages without educational information. Randomization was stratified according to gender, age, and language. The primary outcome was series completion within 12 months. In post hoc analysis, enrollees were compared with concurrent nonenrollees and historical controls.

**Results:**

Overall, 956 parents were enrolled in the study. The precision (475 families) and conventional (481 families) SMS text message arms had similarly high series completion rates (344/475, 72.4% vs 364/481, 75.7%). A total of 42 days after the first dose, two-thirds of families, not initially in the preparation stage, moved to preparation or vaccinated stage. Those in either SMS text message arm had significantly higher completion rates than nonenrollees (708/1503, 47.1% vs 679/1503, 45.17%; *P*<.001). Even after removing those needing only 2 HPV doses, adolescents receiving any SMS text messages had higher completion rates than historical controls (337/2823, 11.93% vs 981/2823, 34.75%; *P*<.001). A population-wide effect was seen from 2014 to 2016, above historical trends.

**Conclusions:**

SMS text message reminders led to timely HPV vaccine series completion in a low-income, urban, minority study population and also led to population-wide effects. Educational information did not provide an added benefit to this population.

**Trial Registration:**

ClinicalTrials.gov NCT02236273; https://clinicaltrials.gov/ct2/show/NCT02236273

## Introduction

### Background

Adolescents, particularly minority adolescents, are not adequately protected against human papillomavirus (HPV) and its potential sequelae, including cancer and genital warts. Despite the highly efficacious vaccine being recommended for all adolescents, completion rates among those who initiate the series are low [1]. Nationwide, only 71.5% of adolescents aged 13-17 years initiate the HPV vaccine series, and half (54.2%) have received all needed doses [1]. Adherence to the recommended HPV vaccine dosing schedule is also exceedingly low; one study found that of 9- to-16-year-olds who had initiated HPV vaccination, only 28% completed the then-recommended 3-dose series within 1 year [2]. Caregiver-decided vaccination delays can significantly contribute to the spread of infectious diseases in adolescents [3]. This is a particularly salient factor to counter for HPV vaccination, as HPV infection not only carries short-term infection risk but also long-term chronic disease risk.

Health information technology interventions that link communication technologies, such as SMS text messaging, with electronic health record data offer low-cost, scalable opportunities to foster vaccination and other preventive care behaviors [4]. Currently, there is a growing body of work supporting the feasibility, acceptability, and efficacy of eHealth and mobile health (mHealth) technologies [5,6], particularly within the realm of adolescent and child health promotion [7,8].

Studies have demonstrated the effectiveness of mHealth SMS text message interventions on vaccination coverage and timeliness at levels in line with other forms of reminder or recall, particularly in low-income populations for whom other forms of reminder recall have been less successful [9-13]. SMS text message reminders have been found to increase HPV vaccine uptake in various populations, particularly in the adolescent cohort [11,14-21]. However, their effect has not been as robust as needed. One potential advantage of SMS text message interventions, which has not been well investigated, is the ability to provide precision messages. Such tailored messages may promote increased engagement with SMS text message reminders and, in turn, positively impact HPV vaccination completion rates.

### Objectives

In this study, we compare the impact of precision SMS text message on HPV vaccine series completion (tailored vaccine health literacy–promoting information targeted to the family’s stage of vaccine decision-making) with conventional SMS text message reminders in a pragmatic randomized trial. The transtheoretical model of behavior change guided the tailoring of SMS text messages.

## Methods

### Overview

This trial was conducted in 4 community health clinics affiliated with the New York-Presbyterian Hospital Ambulatory Care Network and Columbia University between December 2014 and December 2017. These practices provide care for primarily publicly insured Latino patients. The Vaccines for Children program provides free vaccines for nearly all the patients at the study sites, and all the study sites allow walk-ins for second and third HPV vaccine doses without an appointment. All vaccinations given at the study sites are documented in the New York-Presbyterian Hospital immunization registry**,** which extracts information about vaccinations directly from the provider order entry module of the electronic health record, making data accurate for HPV vaccines administered at clinical sites. The registry also synchronizes data with the New York Citywide Immunization Registry (CIR), which is a population-based registry. The New York City Public Health Law requires documentation for all vaccinations administered to those aged ≤18 years old be submitted to CIR [22], which captures >85% of vaccines administered in New York City and 93% of vaccines from the Vaccines for Children program.

Recruitment followed a 2-pronged enrollment process. First, nurses at the study sites provided families with a recruitment card and information sheet. Families interested in being contacted could put a cell phone number on the card, which was then left for the research assistants. Those who did not want to be contacted could also indicate this as such. Of the 547 families from whom a recruitment card was collected, only 9 (1.6%) indicated that they wished not to be contacted. Permission was received to contact the families of all adolescents who received their first HPV vaccine dose at one of the study sites during the enrollment period for whom a card was not left or for whom the nurse did not have time to give a card to assess eligibility and interest.

Parents or legal guardians involved in the study had to meet the following eligibility criteria: (1) have a child aged between 9 and 17 years who received their first HPV vaccine at study clinics, (2) own a cell phone with SMS text message capabilities, (3) have English or Spanish literacy, (4) plan to remain in New York City for the next 12 months, and (5) have not been previously contacted to enroll with a different child. Children down to 9 years were included as the vaccine was licensed down to those aged 9 years. There was no compensation for enrollment in the study.

After each enrollment, the project coordinator used an adaptive electronic randomization algorithm to parallel randomize all participants with a 1:1 allocation ratio stratified by (1) clinic site, (2) adolescent’s gender, (3) adolescent age group (9-14 years vs 15-17 years), and (4) parental language. The scheme was adaptive in that the assignment of each family was to the arm that would keep the allocation ratios in the 4 strata the family belonged to (site, sex, age, and language) closer to 1:1. The statistician and analyst were blinded to the arm assignments.

We designed the messages (*precision* and *conventional*) using information gathered during our previous studies, the relevant literature, and expertise in SMS text messaging, HPV vaccination, adolescent medicine, health literacy, and the community. Messages were then pretested with 20 parents iteratively until no new message changes were made. Parents participating in this pretest received a round trip New York City Metrocard.

**Figure 1:**
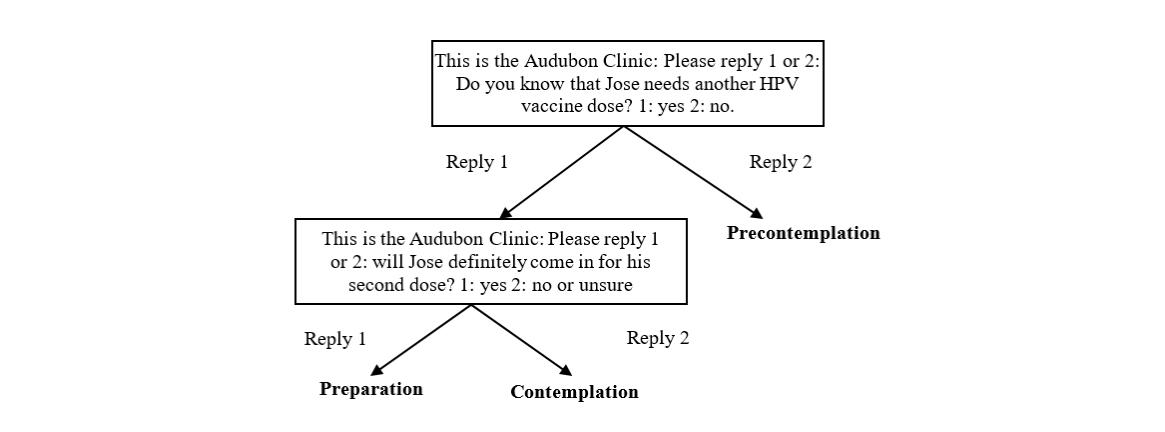
Transtheoretical model stage allocation in the day 21 message. HPV: human papillomavirus.

For families receiving precision SMS text message reminders, an automated, in-house SMS text messaging software program first used a short cascade of questions based on the transtheoretical model to assess the family’s stage of decision-making regarding vaccination (Figure 1).

This was sent on day 21 after administration of the first HPV vaccine dose. On the basis of the person’s response, the platform automatically placed them into the correct stage and proceeded to send educational information targeted at that stage of decision-making. Parents who were in the *precontemplation* stage were unaware that their adolescents needed a second vaccination or when it was due. Those in *contemplation* knew their child or adolescent needed a dose but might still have had questions such as vaccine efficacy, side effects, and safety. Finally, those in *preparation* were planning to have their adolescents continue the vaccination series but might not have known where or when to access care. The program was monitored by the project coordinator.

**Figure 2:**
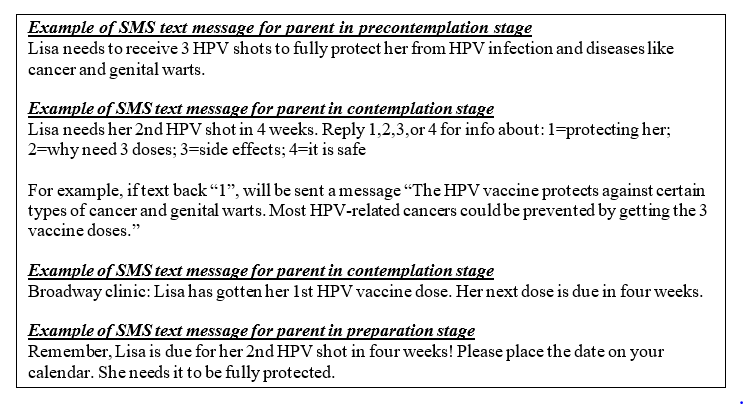
Precision and conventional arm SMS text message examples. HPV: human papillomavirus.

Parents in each stage received different messages (Figure 2). For example, messages for parents in *precontemplation* first notified them that their adolescents were in need of subsequent doses and why those doses were needed, whereas messages for parents in the *contemplation* stage provided information needed to answer any remaining questions they might have had regarding vaccination. For some messages were responsive to user input, such that parents were able to self-tailor the content by texting back indicators for which items they wanted more information about. For those in the *preparation* stage, the messages provided information regarding where and when to walk-in for vaccination. Parents in the other 2 stages also received information regarding where and when to walk-in for vaccination. Families also had 2 additional instances, on days 33 and 40, where their current stage was assessed, if they were not already in the preparation stage. On the basis of responses to these messages, families could switch into a different stage track of messages.

### Conventional SMS Text Message Reminders

Parents not randomized to the precision text message arm received conventional text reminders notifying them when the next dose was due. These messages did not include vaccine health literacy–promoting information and were similar to those used in our previous adolescent studies (Figure 2).

### Message Frequency

Informational messages for both arms began on day 28. This was designed so that, if a family reacted to the message and came in to be vaccinated, it would not be before the 28-day minimal required interval between the first and second dose; the intervention began before the HPV recommendation changed from 3 to 2 doses for younger populations [23]. Subsequent messages were sent on days 35, 42, 49, and 56 post vaccination for both study arms. These dates were chosen because of their proximity to the initial vaccination date; the second dose was due on day 56 (counting from the day the first dose was administered). We selected reminder message send dates at the time they were due and included an additional 1 week (63 days), 2 weeks (70 days), 4 weeks (84 days), and 6 weeks (98 days) *overdue* reminders. This series of 5 messages (days 28, 35, 42, 49, and 56) was selected based on the protocol from our previous trial in which a median of 5 messages was needed for a family to bring a child in for vaccination and was well tolerated by parents with very few stop requests [10]. Booster messages were also sent on days 63, 70, 84, and 98 post vaccination. Once or if an adolescent received the second dose, parents began receiving their next set of messages 28 days before the due date of the next dose, and then followed the same timing as with the second dose messages (days 28, 21, 14, 7, and 0 before the third dose).

Messages were sent in English or Spanish, based on parent preference. On the basis of our previous study [24], which identified parental preferences, messages were designed to include the child’s name and stating that it was being sent on behalf of the clinic. The recipient of the messages was the parent, rather than the child, based on previous preferences elicited in this population. Families stopped receiving messages on their original schedule when the required dose was abstracted from the hospital’s immunization registry, which included synchronized data from the CIR, as described earlier.

In October 2016, the Centers for Disease Control and Prevention (CDC) Advisory Committee on Immunization Practices recommended a change to 2 doses of the HPV vaccine series (0 and 6 months) for those children or adolescents who start the series under the age of 15 years. There was no change in those who started the series when they were aged ≥15 years. In response, the following alterations were made: parents of adolescents <15 years when first vaccinated, who were already enrolled, and had not yet received their second dose, received an SMS text message update informing them that their child now only needed 2 doses 6 months apart and that we would text them when the second dose was due. These parents then received an updated series of messages consistent with the new 2-dose recommendation in terms of both phrasing and timing. Parents of adolescents who either initiated the series (1) at the age of ≥15 years or (2) at <than 15 years but had already received their second dose less than 6 months after the first, stayed on the original protocol because the adolescent was still in need of 3 doses. Any parents of already-enrolled adolescents who were aged <15 years when first vaccinated and received their second dose at least 5 months after their first were considered to have completed the series under the new recommendations. These parents received an SMS text message letting them know that, because their child’s second shot was at least 5 months after the first, they had now completed the series.

### Measures and Analysis

On the basis of previous baseline data, 50.9% (245/481) of patients in the *conventional* group were expected to receive 3 doses in 12 months. With a sample size of 956, we were powered at 80% to detect a 9% difference in completion rate (the primary outcome) between arms to be statistically significant at *P*=.05. The primary outcome measure was timely HPV vaccine series completion within 12 months (operationalized as the receipt of 2 or 3 doses, based on age and enrollment date, and accounting for the 2016 CDC guideline change). Vaccine completion was extracted from local vaccine sources.

HPV vaccine completion rates were compared for all adolescents of participant parents at the end of a 12-month observation period starting at the receipt of their first HPV vaccine dose. All primary analyses were conducted on an intention-to-treat basis. Completion rates in the 2 randomized groups were compared using 2-tailed chi-squared tests at a significance level of *P*<.05. Multiple logistic regressions were performed to assess any potential differences in receipt among demographic groups.

Chi-square tests were conducted as a post hoc analysis to compare both intervention arms with concurrent nonenrollees (n=1503) who received their first vaccine dose during the study period, as well as with historical controls (n=2823; first dose administered 2011-2013). Intervention-arm adolescents who received 2 doses per the new guideline were removed to achieve comparability for the historical analysis. This study was approved by the Columbia University Irving Medical Center Institutional Review Board. Personalized Reminders for Immunization Using Short Messaging is registered with ClinicalTrials.gov with identifier NCT02236273.

### Role of the Funding Source

The Agency for Healthcare Research and Quality funder of the study had no role in the study design, data collection, data analysis, data interpretation, or writing of the report.

## Results

### Overview

**Figure 3:**
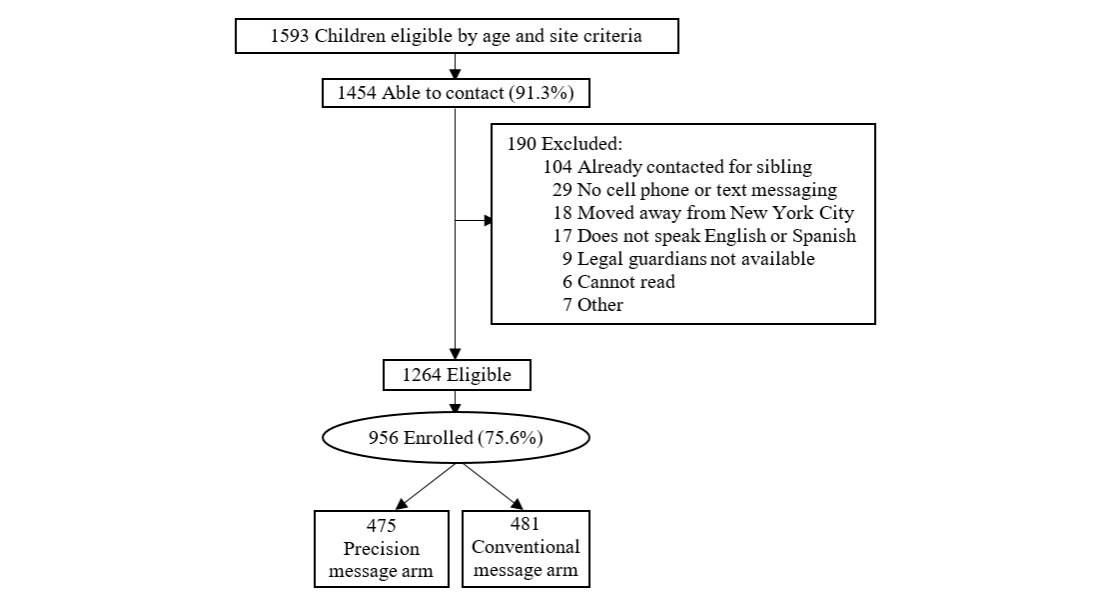
Study enrollment.

In total, we screened 1593 adolescents who received their first HPV vaccine dose at the study sites. The majority (1454/1593, 91.3%) were able to be contacted. Of the contacted patients, only 2% (29/1454) of families did not have a cell phone with SMS text messaging, and 11.1% (161/1454) of were excluded based on other exclusion criteria. Of the eligible families, most (956/1264, 75.63%) were enrolled (Figure 3).

The arms were well-balanced; 481 families were randomized to the usual care arm and 475 to the intervention arm. Most of the adolescents in the enrolled families were aged ≤14 years (880/956, 92.1%). Half of them (478/956, 50%) were female, and most of them (903/956, 94.5%) were publicly insured. Two-thirds (614/956, 64.2%) of parents/caregivers were primarily Spanish speaking, and 59.9% (573/956) had a high school education or less (Table 1).

**Table 1:** Characteristics of the study participants.

Characteristics		Total (n=956), n (%)	Precision message arm (n=475), n (%)	Conventional message arm (n=481), n (%)	*P* value	
**Age (years)**						.95
	<14	880 (92)	437 (92)	443 (92.1)		
	15-17	76 (8)	38 (8)	38 (7.9)		
**Sex**						.56
	Female	478 (50)	242 (51)	236 (49.1)		
**Language**						.88
	Spanish	614 (64.2)	304 (64)	310 (64.5)		
**Site**						.90
	Clinic 1	238 (24.9)	121 (25.5)	117 (24.3)		
	Clinic 2	239 (25)	114 (24)	125 (26)		
	Clinic 3	296 (31)	147 (30.9)	149 (31)		
	Clinic 4	183 (19.1)	93 (19.6)	90 (18.7)		
**Insurance**						.30
	Public	903 (94.5)	445 (93.7)	458 (95.2)		
**Parental education**						.64
	<High school	219 (22.9)	114 (24)	105 (21.9)		
	Finished high school	354 (37.1)	170 (35.8)	184 (38.3)		
	>High school	382 (40)	191 (40.2)	191 (39.8)		

### Movement Through Stages

Overall, 12,000 messages were sent. Of those randomized to the intervention arm, 1 family received their second dose early, and therefore, did not receive second dose messages; a second family requested to stop the program before the messages started. This left 473 of the 475 families randomized to the intervention arm eligible to receive messages. Most families (428/473, 90.5%) in the precision reminder arm received the day 21 message. There were technical issues for 45 families (45/473, 9.5%), as some messages were undelivered because of user service disruption. Of those who received the messages, two-thirds (288/428, 67.3%) of families responded to stage-assessment messages: 52.6% (225/428) were in preparation, 10.3% (44/428) contemplation, and 4.4% (19/428) precontemplation. The remaining families including the 32.7% (140/428) who did not respond and 10.5% (45/428) for whom there were technical difficulties remained in precontemplation.

On day 33, there were 215 families randomized to the precision arm that was either not in preparation or had not yet been vaccinated. Of those, 60% (129/215) responded to either day 33 or 40 messages, 54.4% (117/215) were automatically switched to preparation, 3.7% (8/215) remained in precontemplation, and 1.4% (3/215) moved into contemplation. The remaining stayed in the stage they had been in previously.

By day 42, 72.7% (344/473) of the intervention families were in preparation, with 47.6% (225/473) being there at day 21, and an additional 25.2% (119/473) who moved there through prompts. An additional 34 who had not been in preparation at the beginning of the study had already been vaccinated by day 42, resulting in 79.7% (377/473) either being in preparation or already vaccinated by day 42. Overall, 13.5% (64/473) of families did not respond to the stage questions.

The movement was similar for the third dose. Overall, 336 families randomized to the precision message arm needed a third dose of the vaccine, including those for whom a third dose was needed based on the year and age at first dose. We received replies from half (181/336, 53.9%) of the participants. Of those who were not in the preparation stage at the beginning of the third dose cycle, half were able to be moved into preparation within 2 weeks before the next dose was due.

### Receipt of HPV Vaccination

Of those receiving SMS text messages, Spanish-speaking parents (adjusted rate ratio 1.17, 95% CI 1.08-1.27) had an increased rate of timely completion; ≥15 years old had a decreased completion rate (adjusted rate ratio 0.95, 95% CI 0.93-0.98). No differences existed in terms of sex, education, or insurance.

Overall, both the precision SMS text message (344/473, 72.7%) and conventional (364/481, 75.7%) arms had very high timely series completion rates within 12 months, which did not significantly differ (*P*=.25). We found a significant difference in completion rate for those who responded to the first day 21 intervention messages (n=291) versus those who did not respond (n=153; 219/291, 75.3% vs 100/153, 65.4%; *P*=.04).

**Figure 4:**
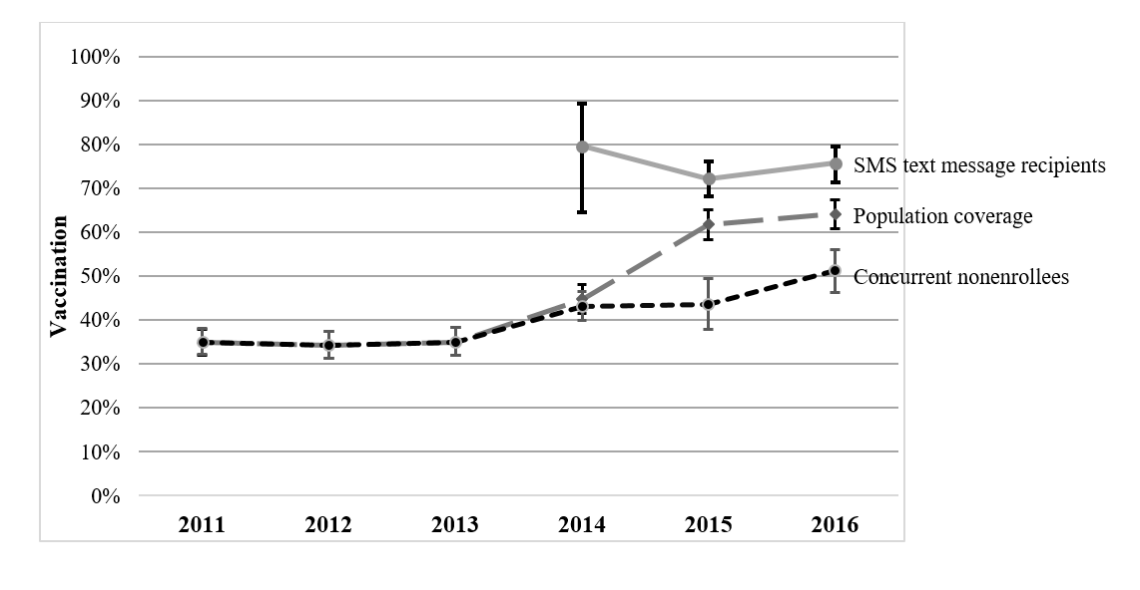
Human papillomavirus vaccine series completion within 12 months of initiation by year of initiation.

In a post hoc analysis, those in either SMS text message arm had a significantly higher completion rate than the nonenrollees. In addition, even after removing those who only needed 2 doses to complete the series, those in the SMS text messaging arms had higher rates than the historical controls (n=2823). Enrollees were more likely to speak Spanish (614/917, 66.9% vs 830/1444, 57.5%; *P*<.001) and were more likely to have a child aged <15 years (880/956, 92.1% vs 1300/1503, 86.49%; *P*<.001) than nonenrollees, but there were no differences in the percentage of children enrolled who were male (479/956, 50.1% vs 795/1503, 52.9%; *P*=.19). In the enrollee comparison to historical controls, those enrolled versus historical controls were less likely to be male (479/956, 50.1% vs 1749/2823, 62%; *P*<.001). There was no difference in the percentage of participants who spoke Spanish (614/917, 66.9% vs 1788/2754, 64.9%; *P*=.35). Ultimately, a population-wide effect on HPV vaccination series completion was seen during the years of the study 2014-2016, above historical trends (Figure 4).

## Discussion

### Principal Findings

In this study, the addition of educational information in SMS text messages targeted to the stage of caregiver vaccine decision-making did not provide additional benefits in this low-income, urban, minority population. However, those receiving SMS text message reminders had more timely series completion than historical controls and nonenrollees. SMS text messaging also led to population-level effects that far exceeded historical trends, illustrating the potential impact of such reminders if implemented. Although ad hoc analyses, the very large differences in completion between enrollees and nonenrollees as well as historical controls adds further support. Together, our findings suggest that SMS text message reminders could be used to increase the likelihood that caregivers will follow through with HPV vaccination series completion and could also be used to combat vaccination delay.

Of those in the intervention group, more individuals who responded to any message on day 21 received the required doses than those who did not. However, in this study population, education information in the SMS text messages did not provide additional benefits over conventional SMS text messages without educational information. Studies have shown that prolonged exposure to similarly themed messages may lead to *reactance* or active resistance against the health behavior the health message advocates [25,26]. In addition, for parents with an unfavorable attitude toward vaccines, educational interventions have been found to reduce the intention to vaccinate [27]. For the subset of our patient population who failed to interact with the SMS text messages, additional educational messages may have acted as a mental deterrent to bringing in their children for subsequent doses. It is possible that for certain populations, changing to a different modality is needed. These messages were based on formative work and pretesting with patients and their families, which is critical in the development of mHealth interventions [28-31]; however, future work should also potentially include an intentional exploration with the target populations of unintended impacts of messages.

Despite this finding, our study demonstrates how SMS text message reminders could interrupt a common pathway to vaccination delay and vaccine series incompletion. Vaccination delay, particularly for HPV vaccination, is often studied as an active decision by caregivers [32]; however, given the increase in timeliness and the lack of impact of targeted vaccine-readiness information shows that delay is often not an active decision but rather a circumstantial effect based on other factors (eg, forgetfulness) that can be mitigated with timely reminders. SMS text message reminders work as a *call to action* and help prompt caregivers that would otherwise vaccinate their children but may fail to bring their child back for vaccination because of other factors. Further research could be conducted to explore the benefit of this technology for direct youth use, as an increasing number of health interventions are targeted at adolescent self-use to encourage increases in their health autonomy [33].

In this study, we also demonstrated the ability to use SMS text messages to assess a family’s stage of vaccine decision-making and move them along the stages of the transtheoretical model. Most families (409/473, 86.5%) responded to at least 1 message prompt, and two-thirds of families who were not in preparation at the first assessment were either in preparation or vaccinated by 42 days after the first dose. Similarly, half of the families of adolescents who needed 3 doses who were not in preparation at the first assessment for that dose were either in preparation or vaccinated by the time the third dose was due. Although ultimately such an in-depth, precision intervention may not have been needed for this population, it does lay the foundation for using SMS text messaging both to assess a person’s stage of decision-making and to intervene to move them through to a possible behavior change. Several SMS text messaging interventions that have targeted stages of change have been tested and found to be effective in encouraging health behavior change, namely with physical activity [34-36], smoking cessation [37,38], and diabetes care management [39]. However, many of these studies were conducted internationally, and none have addressed HPV vaccine uptake. Our study contributes to the growing body of knowledge on SMS text messages targeted to the stage of change and presents a novel understanding of SMS text message efficacy in increasing HPV vaccination completion in adolescents. These findings may be particularly applicable given the increased levels of vaccine hesitancy in caregivers in the wake of acute COVID-19 activity. Although app-based interventions offer a number of benefits, they require users to have higher levels of technological literacy than SMS text message–based interventions. SMS text messages are preset on mobile phones and require virtually no instruction for use when receiving messages outside general literacy. The results of this study, along with prior vaccine SMS text message reminder research, underscore the sustained role SMS text messaging can still play in providing a digital precision SMS text message health approach to behavioral interventions, even in the modern mobile use landscape.

During this study, the CDC changed their recommendations for the number of doses of HPV vaccine needed by adolescents who initiated the series before the age of 15 years. An unintended benefit of this study was the demonstration of the ability of SMS text messages to facilitate rapid communication with families to inform them of the CDC schedule changes. Such ability extends the possibilities for health care providers to notify families when they need them to either take or not take a certain action. If sites had to call families to tell them not to come in, it would have required extensive personnel time. Conversely, it would have been frustrating to families if they had not been notified and had showed up too early. This real-time notification can be beneficial for both health care providers and public health practitioners. Investigating modalities and best practices of remote pediatric clinic communication with caregivers is particularly needed as we rethink health care communication and adolescent care in the wake of the height of the COVID-19 pandemic [40,41].

### Limitations

Our study has several limitations. This study took place in a single medical system that serves a primarily low-income, Latino, urban community, who may be particularly sensitive to SMS text message interventions. These findings may not be generalizable to other settings. Recommendations also changed during the intervention period. Most of the study population received the first dose before the new CDC guidelines were implemented, when 3 doses were needed. However, these were accounted for in the analyses. In addition, vaccine administration has been underreported. However, all administered vaccines are documented in the electronic health record, including synchronization with the New York CIR, which has an excellent capture rate. Therefore, underreporting of vaccinations is likely low; underreporting would also have affected the intervention and usual care groups similarly.

### Conclusions

Despite these aspects of the study, our findings lend strength to the growing body of evidence showing that mHealth or eHealth interventions such as SMS text message reminders can be used to tangibly promote child and adolescent health [40], particularly in the realm of HPV vaccination, in which outcomes are consistently substandard to national goals. We also demonstrate the efficacy of SMS text message reminders in a low-income, tight knit, and connected minority community, which helps answer the call to improve upon digital approaches that “address disparities in access to care related to race and ethnicity, socioeconomic status” [40]. SMS text message reminders function as an accessible, easy-to-use, low-cost remote intervention that can be rapidly deployed, although information detailing the economic impact and cost-effectiveness of these interventions should be evaluated in future studies.

SMS text message reminders led to timely HPV vaccine series completion in our study population, which led to population-wide effects. Although education information did not provide added benefit in this very responsive population, we also demonstrated the feasibility of using SMS text messages to both identify a family’s stage of vaccine decision-making, move them further down the pathway to behavior change, and possibly decrease HPV vaccine delay. In the face of health information changes, SMS text messages also helped facilitate real-time and remote communication of these changes to caregivers, which is needed in our post pandemic clinical pediatric landscape.

## Abbreviations

CDC: Centers for Disease Control and Prevention
CIR: Citywide Immunization Registry
HPV: human papillomavirus
mHealth: mobile health
